# Potential of Disease-Modifying Anti-Rheumatic Drugs to Limit Abdominal Aortic Aneurysm Growth

**DOI:** 10.3390/biomedicines10102409

**Published:** 2022-09-26

**Authors:** Shivshankar Thanigaimani, Muhammad Ibrahim, Jonathan Golledge

**Affiliations:** 1 The Queensland Research Centre for Peripheral Vascular Disease (QRC-PVD), College of Medicine and Dentistry, James Cook University, Townsville, QLD 4811, Australia; 2 The Australian Institute of Tropical Health and Medicine, James Cook University, Townsville, QLD 4811, Australia; 3 The Department of Vascular and Endovascular Surgery, Townsville University Hospital, Townsville, QLD 4811, Australia

**Keywords:** abdominal aortic aneurysm, disease-modifying anti-rheumatic drugs, tumour necrosis factor-α, interleukin

## Abstract

Inflammation is strongly implicated in the pathogenesis of abdominal aortic aneurysms (AAA). This review examined the potential role of biologic disease-modifying anti-rheumatic drugs (bDMARDs) as repurposed drugs for treating AAA. Published evidence from clinical and preclinical studies was examined. Findings from animal models suggested that a deficiency or inhibition of tumour necrosis factor-α (TNF-α) (standard mean difference (SMD): −8.37, 95% confidence interval (CI): −9.92, −6.82), interleukin (IL)-6 (SMD: −1.44, 95% CI: −2.85, −0.04) and IL-17 (SMD: −3.36, 95% CI: −4.21, −2.50) led to a significantly smaller AAA diameter compared to controls. Human AAA tissue samples had significantly increased TNF-α (SMD: 1.68, 95% CI: 0.87, 2.49), IL-1β (SMD: 1.93, 95% CI: 1.08, 2.79), IL-6 (SMD: 2.56, 95% CI: 1.79, 3.33) and IL-17 (SMD: 6.28, 95% CI: 3.57, 8.99) levels compared to non-AAA controls. In human serum, TNF-α (SMD: 1.11, 95% CI: 0.25, 1.97) and IL-6 (SMD: 1.42, 95% CI: 0.91, 1.92) levels were significantly elevated compared to non-AAA controls. These findings implicate TNF-α, IL-17 and IL-6 in AAA pathogenesis. Randomised controlled trials testing the value of bDMARDs in limiting AAA growth may be warranted.

## 1. Introduction

Abdominal aortic aneurysms (AAA) are a common vascular condition of older adults that are estimated to be responsible for about 200,000 deaths per year due to AAA rupture [1]. AAA is usually asymptomatic and identified during routine abdominal imaging, such as ultrasound and computed tomographic angiography [2]. The only current treatment is AAA repair by open or endovascular surgery, but this is only indicated in people with large (≥55 mm in men and ≥50 mm in women) asymptomatic or symptomatic aneurysms [3,4]. Small, asymptomatic AAAs are managed by imaging surveillance, but up to 70% of AAAs expand to a size at which surgical repair is considered [5]. Drugs are needed that effectively slow AAA growth [6,7,8].

Experimental and human observational studies suggest an important role of inflammation in AAA pathogenesis, denoting the potential benefit of anti-inflammatory medications in limiting AAA growth [6]. The role of inflammation in aortic pathology has previously been reviewed in detail [9,10]. Disease-modifying anti-rheumatic drugs (DMARDs) are a class of drugs that block inflammatory cytokines and have been approved for use in several inflammatory conditions, including rheumatoid arthritis, psoriasis, ankylosing spondylitis, systemic lupus erythematosus, Sjogren’s syndrome and multiple sclerosis [11]. Three major categories of DMARDs have been developed that block tumor necrosis factor (TNF) and interleukins (IL). TNF and IL inhibitors belong to the biologic DMARDs (bDMARDs) category. This review first details commonly used DMARDs, then critically reviews evidence from preclinical models and people with AAAs of which TNF and IL are involved in pathogenesis. Finally, there is a discussion of the data on the safety of these agents in older people, such as those with AAA. The intent of this review is to explore the potential of using bDMARDs as repurposed drugs for limiting AAA progression.

## 2. Disease-Modifying Anti-Rheumatic Drugs

bDMARDs were developed because many patients with inflammatory diseases were unable to attain optimal responses to conventional DMARDs (e.g., Methotrexate, Leflunomide, Hydroxychloroquine and Sulfasalazine). An overview of the currently available bDMARDs is provided in Table 1, and detailed information on these drugs has been reviewed elsewhere [11,12].

## 3. Evidence from Animal Models for a Role of TNF and IL in AAA Pathogenesis

### 3.1. Animal Studies Investigating the Effect of Blocking TNF-α on AAA Development and Growth

Three previous studies in two different rodent models have reported that inhibiting TNF-α reduced the size of the AAA that developed compared to controls [28,29,30]. The method of downregulating TNF-α varied. One study tested two different methods of blocking TNF-α, namely gene knockout and a monoclonal antibody, within the calcium chloride aneurysm model [28]. Mice deficient in TNF-α developed smaller aneurysms than TNF-α sufficient controls in response to peri-aortic calcium chloride application [28]. This study also examined the effect of blocking TNF-α using a monoclonal antibody against TNF-α (infliximab) [28]. It was reported that the administration of infliximab weekly beginning from the day of calcium chloride application to 6 weeks after AAA induction significantly inhibited infra-renal aortic expansion [28]. TNF-α is generated from its precursor through the action of TNF-α converting enzyme (TACE). An inducible deficiency of TACE was reported to downregulate TNF-α and significantly reduce aortic expansion induced by the peri-aortic application of calcium chloride [29]. TNF binding protein (TNF-BP) is a dimer of the soluble form of the TNF receptor that inhibits TNF-α [30]. The administration of TNF-BP to Wistar rats has been reported to significantly inhibit aortic expansion over 6 days after intra-aortic elastase perfusion [30].

**Figure 1:**
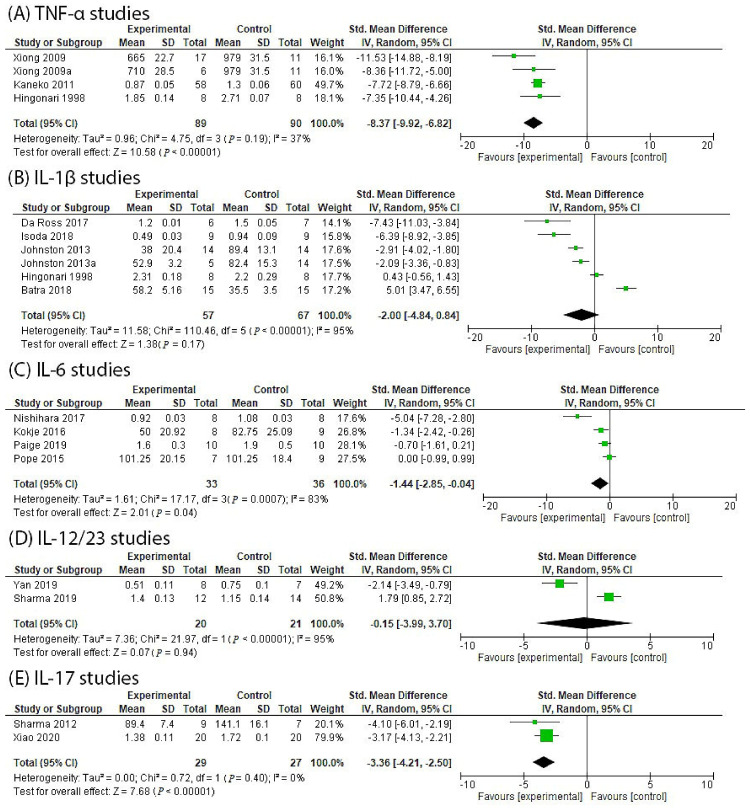
Meta-analysis testing the effect of blocking TNF or IL in rodent models of AAA. (**A**) TNF-α inhibition significantly reduced AAA diameter [28,30,31]; (**B**) IL-1β inhibition did not significantly reduce AAA diameter [30,32,33,34,35]; (**C**) IL-6 inhibition significantly reduced AAA diameter [36,37,38,39]; (**D**) IL-12/23 inhibition did not significantly reduce AAA diameter [40,41]; (**E**) IL-17 inhibition significantly reduced AAA diameter [42,43]. Note: Meta-analyses were performed only when a minimum of two relevant studies were identified. TNF, tumour necrosis factor; IL, interleukin; AAA, abdominal aortic aneurysm.

A meta-analysis including all four investigations from these three published studies, including a total of 89 experimental and 90 control rodents, found that a deficiency or inhibition of TNF-α led to a significantly smaller AAA diameter in experimental animals compared to controls with a large effect size and moderate heterogeneity (Figure 1A). A deficiency or inhibition of TNF-α was reported to limit AAA severity by multiple mechanisms in these studies, including reducing pro-inflammatory cytokines and adhesion molecule expression, limiting macrophage infiltration and reducing vascular endothelial growth factor (VEGF-A) expression, neoangiogenesis, matrix metalloproteinase (MMP) activity and extracellular matrix (ECM) remodeling (Table 2) [28,29].

The quality of the animal studies was assessed using a modified version of the Animal Research: Reporting of In Vivo Experiments (ARRIVE) guideline criteria (Table 3). All studies reported aortic diameter change, but only one study explained the methods used to measure aortic diameter and reported reproducibility [30]. The studies neither randomized the animals to specific groups, nor blinded the assessor during outcome assessment (Table 3).

### 3.2. Animal Studies Investigating the Effect of Blocking IL-1β or IL-1R on AAA Development and Growth

IL-1β and IL-1 receptor (IL-1R) belong to the IL-1 family of ligands and receptors that heterodimerize upon binding and modify the immune response [47,48]. Studies testing blocking IL-1β or IL-1R were analysed together due to their biological and functional relationship. A total of six previous studies testing whether IL-1β or IL-1R inhibition reduced AAA size in three different rodent models were identified [30,32,33,34,35,44]. Four studies reported that inhibition of IL-1β or the IL-1R in AAA experimental models developed using elastase perfusion or angiotensin II infusion resulted in smaller AAA size as compared to control mice [33,34,35,44]. In contrast, one study that tested the effect of IL-1β deficiency in the calcium chloride model reported significantly larger AAA size six weeks after aneurysm induction compared to controls [32]. Another study tested an IL-1R antagonist (IL-1Ra) in the elastase perfusion model and reported no significant effect on AAA size [30]. Two studies reported that blocking IL-1β or IL-1R via gene knockout significantly reduced the mean AAA size 14 days after elastase perfusion [35,44]. In one study, administration of the IL-1β monoclonal antibody in mice deficient in IL1Ra (and therefore primed to the action of IL-1β) during AAA induction by angiotensin II infusion significantly reduced AAA size after 4 weeks [34]. In another study, IL-1β monoclonal antibody administration in mice with a germline deficiency of IL1-R1 and a vascular smooth muscle cell-specific deficiency of Mothers against decapentaplegic homolog 4 (SMAD4) developed smaller AAAs during 16 weeks of angiotensin II infusion in comparison to controls [33]. On the other hand, the administration of anakinra, an IL-1R antagonist, at a dose of 100mg/kg/day and commencing up to 7 days after aneurysm induction by an aortic perfusion of elastase, resulted in significant protection against aneurysm growth over 21 days [35]. IL-1β was reported to contribute to aortic inflammation by triggering ceramide synthesis within neutrophils and inducing neutrophil extracellular traps (NETosis) that promoted AAA formation (see Table 2). It is noteworthy that IL-1β deficiency created by gene knockout attenuated aneurysm development in the elastase perfusion [35] but not the calcium chloride model [32]. This highlights disparity in the mechanisms involved in AAA development between the different animal models.

**Figure 2:**
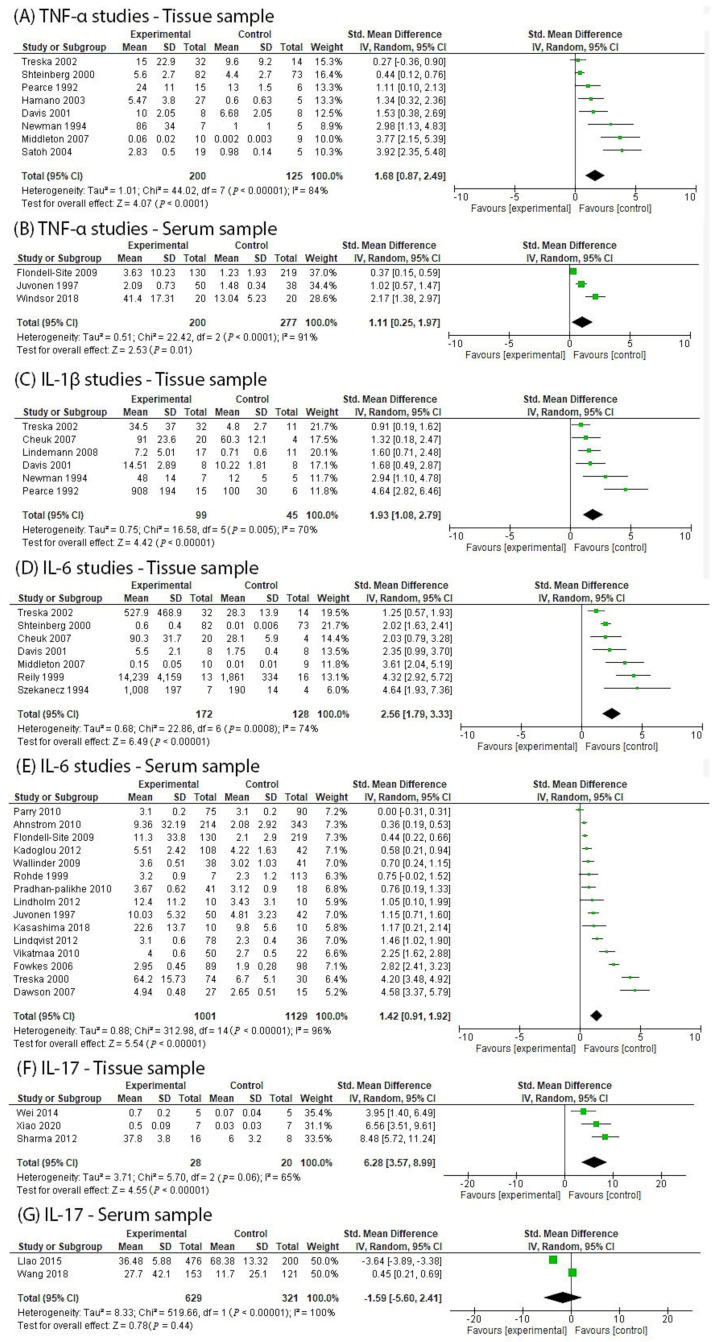
Meta-analysis comparing the expression levels of TNF and IL in human participants with AAA and controls. (**A**) Aortic tissue expression of TNF-α was significantly higher in AAA cases than controls [49,50,51,52,53,54,55,56]. (**B**) Serum concentration of TNF-α was significantly higher in AAA cases than controls [57,58,59]. (**C**) Aortic tissue expression of IL-1β was significantly higher in AAA cases than controls [49,52,53,56,60,61]. (**D**) Aortic tissue expression of IL-6 was significantly higher in AAA cases than in controls [49,51,55,56,60,62,63]. (**E**) Serum concentration of IL-6 was significantly higher in AAA cases than in controls [58,59,64,65,66,67,68,69,70,71,72,73,74,75,76]. (**F**) Aortic tissue expression of IL-17 was significantly higher in AAA cases than in controls [42,43,77]. (**G**) Serum concentration of IL-17 was similar in AAA cases and controls [78,79]. Note: Meta-analyses were performed only when a minimum of two relevant studies was identified. TNF, tumour necrosis factor; IL, interleukin; AAA, abdominal aortic aneurysm.

One study used two different methods to block IL-1R including gene knockout and an IL-1R antagonist, and these investigations were considered separately for the meta-analysis [35]. Overall, a meta-analysis of all the included studies (57 experimental and 67 control mice) suggested no significant effect of IL-1β or IL-1R inhibition on AAA diameter (Figure 2). A high heterogeneity between the included studies was noted (Figure 1B). These results were particularly influenced by one study that reported larger aneurysms in IL-1β-deficient mice. Further investigation suggested that an increased aortic diameter in IL-1β-deficient mice was due to differential macrophage response to IL-1β deletion as compared to TNF-α deletion [32]. Quality assessment showed that all studies reported the methods used to measure the aortic diameter; however, only one study reported reproducibility data and that outcome assessors were blinded to group allocation [35]. None of the included studies randomized the animals during allocation to specific groups (see Table 3).

### 3.3. Animal Studies Investigating the Effect of Blocking IL-6 or IL-6R on AAA Development and Growth

The cytokine IL-6 homodimerizes with its membrane-bound receptor IL-6R to exert its pro-immune functions [80]. Studies that blocked either IL-6 or IL-6R were grouped together for analysis. Four studies testing the effect of IL-6 or IL-6R inhibition in two different aneurysm models were identified [36,37,38,39]. Two studies reported that significantly smaller aneurysms developed after an IL-6 blocking antibody was administered in the elastase perfusion model [36] and an IL-6R blocking antibody was given in the calcium chloride model [37]. When IL-6 was neutralized by administering an antibody (4mg/kg intraperitoneal injection) one day prior to AAA induction, aortic rupture was promoted in greater than 40% of mice within 7 days [36]. Remarkably, aortic ruptures were abolished and aneurysm growth slowed when the antibody administration was initiated three days after AAA induction commenced. This suggests that IL-6 may have a different role in AAA pathogenesis at distinct stages of AAA development in the elastase model [36]. Within the calcium chloride model, tail vein administration of 2 mg of an IL-6R blocking antibody, commencing one day prior to aneurysm induction and repeated at a dose of 0.25 mg intraperitoneally every week, reduced the size of the aneurysm that developed after 6 weeks compared to controls [37]. Another study reported that IL-6 deficiency did not significantly influence the size of the aneurysm induced by elastase perfusion [39]. Similarly, in mice models in which AAA was induced with a subcutaneous infusion of angiotensin II and an injection of an anti-TGFβ antibody, a blockade of the IL-6R-using antibody administered three times a week significantly reduced the aneurysm rupture rate [38].

Overall, a meta-analysis of all the included studies involving 33 experimental and 36 control mice suggested that the blockade of IL-6 or IL-6R significantly reduced AAA size (Figure 1C). A large effect size and a large degree of statistical heterogeneity were noted. Quality assessment showed that all studies reported the methods used to measure aortic diameter; however, no studies performed reproducibility tests for aortic diameter measurements (see Table 3). Only one study blinded the assessor during outcome assessment [36].

### 3.4. Animal Studies Investigating the Effect of Blocking IL-12 or IL-23 on AAA Development and Growth

Ustekinumab, a bDMARD, is an IgG1 humanized monoclonal antibody directed against a common p40 subunit of IL12 and IL-23 [81]. The drug binds to the p40 subunit and neutralizes both IL-12- and IL-23-mediated intracellular responses [81]. The p40 subunit pairs with the p19 subunit of IL-23 and signals through IL-12Rβ1 and IL-23R, and therefore is equally important in the actions of IL-12 and IL-23. Given their shared subunits and intracellular pathways, both IL12 and IL23 were considered together for analysis within this review. Two studies tested the effect of IL-12 or IL-23 gene deficiency on aneurysm size in two different experimental mice models and reported contrasting results [40,41]. Germline IL-12p40 deficiency was reported to promote the formation of larger aneurysms within the angiotensin II infusion model through the promotion of aortic recruitment of M2-like macrophages [40]. In another study, an intraperitoneal injection of IL-12p40 or IL-23p19 monoclonal blocking antibody (250 μg) on days 3 and 8 after AAA induction by the aortic perfusion of elastase resulted in significant protection from aneurysm growth [41]. An injection of IL-12/23 blocking antibody was reported to suppress macrophage expansion in the elastase model [41], but germline IL-12p40 deficiency promoted macrophage recruitment within the angiotensin II infusion model [40]. A similar disparity in the effect of IL-β1 has also been reported in different AAA models [35]. IL-β1 inhibition in the elastase perfusion model has been reported to suppress macrophage recruitment, whereas in other models, it has been shown to promote macrophage recruitment. A number of varying factors, including the use of knockout mice versus a specific antibody, model type, time point of the intervention initiation and different laboratory settings, may have caused these discrepancies and needs to be controlled for more clearly in the future. Further investigation of the effect of ILs on macrophage activation in the different aneurysm models is needed to understand the potential value of bDMARDs.

Overall, a meta-analysis of these studies including 20 experimental and 21 control mice suggested that a blockade of IL12/23 did not affect AAA diameter (Figure 1D). Quality assessment showed that both studies reported the methods employed for measurements of aortic size, but neither of them performed reproducibility tests for the aortic size measurement protocol. One study blinded the assessor during outcome assessment [41] (see Table 3).

### 3.5. Animal Studies Investigating the Effect of Blocking IL-17 on AAA Development and Growth

IL-17 is a family of cytokines that play a key role in the control of the immune response, of which IL-17A (IL-17 subtype-A) is the most well-studied [82]. Recently, IL-17A blocking agents have been approved for the treatment of moderate-to-severe plaque psoriasis [82]. The terms IL-17 and IL-17A are often used interchangeably; therefore, studies investigating IL-17 or IL-17A were considered together and the term IL-17 was used. Two studies tested the effect of inhibiting IL-17 on aneurysm size in the angiotensin II and elastase perfusion models [42,43]. Both studies reported a significant reduction in AAA size in experimental mice as compared to controls. One study reported that IL-17 deficiency reduced the size of AAA that developed 14 days after elastase perfusion compared to controls [42]. In the second study, AAAs were initiated with angiotensin II infusion, and on the same day, mice were given 3μg/kg intraperitoneal injection of small interfering RNA (siRNA) targeting IL-17, and they were monitored weekly for 28 days [43]. Administering IL-17 siRNA reduced aneurysm development, which was reflected in significantly smaller AAA diameters on day 28 in experimental mice by comparison to controls [43]. IL-17 downregulation was suggested to limit aneurysm development by reducing VEGF expression via the Janus kinase/signal transducer and activator of the transcription (JAK/STAT) pathway, in addition to suppressing MMP-2 and MMP-9 expression levels.

Overall, meta-analysis of these studies using 29 experimental and 27 control animals suggested that IL-17 inhibition led to a significantly smaller AAA diameter in experimental animals compared to controls, with a large effect size and low heterogeneity (Figure 1E). Quality assessment showed that neither study randomized the animals to different groups, performed reproducibility tests for aortic diameter measurements or blinded the assessors during outcome measurements (see Table 3).

## 4. Evidence from Human Studies for a Role of TNF and IL in AAA Pathogenesis

### 4.1. Human Studies Investigating TNF-α in AAA Participants

A total of ten studies investigating the levels of TNF-α in aortic tissue samples from 223 AAA cases and 140 controls were identified (Table 4) [49,50,51,52,53,54,55,56,61,83]. Seven studies matched 141 AAA cases and 67 controls for age and sex. One study included AAA patients whose median age was reported to be significantly higher than the control group [55], and two studies did not report the age of the patients studied [50,52]. The control samples used were from a variety of sources, including organ donors or cadavers [50,51,56,61], people with athero-occlusive disease [49,52,53,55] in four studies each and patients with non-cardiovascular diseases or other comorbidities in two studies [54,83]. Three studies measured TNF-α expression via enzyme-linked immunosorbent assay (ELISA) [49,52,53], three used reverse transcriptase polymerase chain reaction (RT-PCR) [54,55,61] and two used immunohistochemistry [50,56]. An antibody-based protein array [51] or illumina microarray [83] were each used in one study. Nine studies reported significantly higher TNF-α levels in AAA samples as compared to controls [49,50,51,52,54,55,56,61,83]. One study reported statistically similar TNF-α levels in AAA tissue samples compared to controls [53]. A pooled analysis of available data from these studies with 200 AAA cases and 125 controls showed significantly higher TNF-α levels in AAA tissue samples, as compared to controls with high statistical heterogeneity and a large effect size (Figure 2A). Quality assessment suggested a high risk of bias for all ten studies (Table 5). None of the studies reported rationales for the sample sizes used or adjusted analyses for comorbidities. Furthermore, none of the studies reported imaging the cases or controls.

Three studies measured TNF-α expression levels in serum samples from 200 AAA cases and compared the results with 277 age- and sex-matched controls (Table 6). Control serum samples were collected either from patients whose coronary angiography results were normal [59] or from healthy males [57,58]. All three studies used an immunoassay to measure TNF-α and reported significantly higher levels in AAA cases as compared to controls. A pooled analysis suggested that circulating TNF-α concentrations were significantly higher in people with AAA, with a large effect size and a high degree of statistical heterogeneity (Figure 2B). Quality assessment suggested a high risk of bias in all three studies (Table 5). Rationales for sample sizes were provided for two studies [57,58], but not the third [59]. All studies reported ultrasound imaging performed for AAA cases and controls; however, none of them blinded the assessor during cytokine measurements.

### 4.2. Human Studies Investigating IL-1β in AAA Participants

A total of nine studies investigating IL-1β in 125 AAA tissue samples and compared with 74 controls were identified [49,51,52,53,56,60,61,83,84]. Eight studies matched 118 AAA cases and 69 controls for age [49,51,53,56,60,61,83,84], and one study did not report the age of the patients studied [52]. Seven studies matched 103 AAA cases and 63 controls for sex [49,51,56,60,61,83,84], and two studies did not report the sex of the patients [52,53]. All studies used control samples from a variety of sources including cadaveric donors or organ donors [51,52,53,56,60,83], athero-occlusive disease patients [49,61] or relatively non-diseased aneurysm neck [84]. For IL-1β measurements, four studies used ELISA [49,52,53,60] and two studies used microarrays [83,84]. A protein-based array [51], RT-PCR [61] or immunohistochemistry [56] were used in one study each. Eight studies reported significantly higher IL-1β expression levels in AAA samples as compared to controls. One study reported similar levels of IL-1β in AAA and control samples [60]. A pooled analysis of available data from these studies including 99 AAA cases and 45 controls showed significantly higher IL-1β levels in AAA samples as compared to controls, with a large effect size and a large degree of statistical heterogeneity (Figure 2C). Quality assessment suggested a high risk of bias for all nine studies (Table 6). None of the studies reported rationales for the sample sizes used or adjusted analyses for comorbidities. Furthermore, none of the studies reported performing imaging of the AAA cases or controls.

One study reported IL-1β levels in serum samples from 50 AAA cases and compared these with 42 age- and sex-matched controls [59]. Significantly higher IL-1β levels were reported in AAA cases by comparison with controls measured with a solid-phase radio-immunoassay [59]. Serum samples from controls were collected from patients whose coronary angiogram results were normal (Table 5). Pooled analysis was not possible, as only one study was identified. Quality assessment suggested a high risk of bias (Table 6). A rationale for the sample size was not provided and assessors were not blinded during cytokine measurements; however, ultrasound imaging of participants was performed.

Based on this evidence that IL-1β may play a role in AAA pathogenesis, a randomized placebo-controlled trial was designed to test the effect of IL-1β neutralization on AAA growth. The trial was stopped prematurely due to perceived futility after only 64 patients were randomized and a total of only 43 patients completed the study. Canakinumab (150 mg), or placebo, was administered subcutaneously once per month for one year. This trial showed similar aneurysm growth in both treatment and control groups, but was undoubtedly underpowered to test any conceivable effect and therefore the effect of IL-1β neutralization on AAA growth remains unclear [86].

### 4.3. Human Studies Investigating IL-6 in AAA Participants

IL-6 is one of the central coordinators of the inflammatory response that renders its actions via both *cis-* and *trans*-signaling [87]. The classical *cis*-signaling occurs through a G-protein-coupled receptor mechanism, resulting in an autocrine effect. *Trans*-signaling occurs through the circulating form of the IL-6 receptor (sIL6-R), resulting in a paracrine effect [87].

A total of eight studies investigated the aortic tissue levels of IL-6 from 182 AAA cases, and 135 controls were identified [49,51,55,56,60,62,63,84]. Six studies matched 159 AAA cases against 109 controls for age and sex [49,51,55,56,60,84]. Two studies did not report the age and sex of the included patients [62,63]. Control aortic samples were obtained from organ donors [51,56,60,63], athero-occlusive disease [49,55,62] or relatively non-diseased aneurysm neck [84]. Four studies measured IL-6 using ELISA [49,60,62,63], and one study each used immunohistochemistry [56], RT-PCR [55], an antibody-based protein array [51] or a gene microarray [84]. Seven studies reported a significantly higher IL-6 expression in AAA samples by comparison with controls [49,51,55,56,62,63,84]. One study reported no statistically significant difference [60].

A pooled analysis including 172 AAA cases and 128 controls found significantly higher IL-6 levels in AAA cases as compared to controls (Figure 2D). Quality assessment suggested a high risk of bias for all eight studies (Table 6). None of the studies reported rationales for the sample sizes used or adjusted analyses for comorbidities. Furthermore, none of the studies reported imaging the cases or controls.

A prior meta-analysis of 13 studies involving 1029 AAA cases and 924 controls suggested higher circulating levels of IL-6 within AAA patients than controls [88]. An updated search identified 16 studies involving 1254 AAA cases and 1149 controls that further confirmed the results from previous meta-analysis [58,59,64,65,66,67,68,69,70,71,72,73,74,75,76,85]. Fourteen studies matched 933 AAA cases and 1105 controls for age [58,59,64,65,66,67,68,69,70,71,73,75,76,85] and thirteen studies matched 923 AAA cases and 1095 controls for sex [58,59,64,65,66,67,68,70,71,73,75,76,85]. One study did not report the age or sex of the included patients [72]. Control patients were from different sources, including those undergoing diagnostic or interventional coronary angiograms [65], those with normal coronary angiogram results [59], those newly referred to vascular, surgical and urology outpatient clinics with <30 mm aortic diameter [71], those scheduled for hip replacement surgery [69], those referred for transthoracic echocardiogram [76], healthy individuals or those with normal infra-renal aortic diameter [58,64,66,70,73,75,85], autopsy cases with no or slight atherosclerotic aorta without dilatation [68], stable coronary artery disease [67] or athero-occlusive disease [72,74]. All studies used ELISA to measure IL-6 levels. Thirteen studies reported significantly higher levels of IL-6 in AAA cases by comparison with controls [58,59,64,65,66,67,68,69,70,73,74,76,85] (Table 5). Three studies reported similar concentrations in cases and controls [71,72,75]. A pooled analysis of available data from 1001 AAA cases and 1129 controls showed significantly higher IL-6 levels in aneurysm patients as compared to controls (Figure 2E). Quality assessment suggested a high risk of bias for all sixteen studies (Table 6). Two studies reported rationales for the sample sizes used [58,71], and one study adjusted analyses for comorbidities [71]. Only one study blinded the assessor during IL-6 measurements [65]. Furthermore, thirteen studies reported imaging the cases and controls [58,59,64,65,66,67,68,70,71,73,75,76,85].

A meta-analysis of seven genetic studies including 869 AAA cases and 851 controls used the Mendelian randomization approach to demonstrate that a single nucleotide polymorphism (SNP) of the Asp358Ala allele variant (rs2228145) in the IL-6R gene was associated with a lower risk of AAA. This SNP is believed to be associated with a reduced expression of downstream targets in response to IL-6 stimulation. This analysis suggested that IL-6 was likely to play a causal role in AAA [89]. Based on this evidence, the association between the IL6R-Asp358Ala variant and annual change in AAA diameter was recently estimated using a linear mixed-effects regression model [38]. The study included 2863 AAA patients from nine prospective cohorts. After adjusting for age and sex, modeling data showed a yearly -0.06 (-0.18 to 0.06) mm change in AAA growth per copy of the minor allele [38]. Unfortunately, the study was not adequately powered to test the association of genetic variation in the IL6R with AAA growth.

### 4.4. Human Studies Investigating IL-12/23 in AAA Participants

Three studies investigating the effect of IL-12/23 in aortic tissue samples from 33 AAA cases and 27 age- and sex-matched controls were identified [51,61,83]. Control aortic samples were collected from organ donors [51,83] or patients with aortic athero-occlusive disease [61]. Two studies reported significantly higher IL-12/23 expression levels in AAA cases by comparison with controls when measured using a microarray [83] or antibody-based protein array [51]. One study could not detect IL-12/23 levels in samples using RT-PCR [61]. Pooled analysis was not possible due to very low, undetectable concentrations [61] in one study and the lack of available data [83] from another study (Table 4). Quality assessment suggested a high risk of bias for all three studies (Table 6). None of the studies reported rationales for the sample sizes used, adjusted analyses for comorbidities or blinded the assessor during IL-12/23 measurements.

### 4.5. Human Studies Investigating IL-17 in AAA Participants

Three studies investigating IL-17 expression levels in aortic tissue samples in 28 AAA patients and 20 age- and sex-matched controls were identified [42,43,77]. None of the three studies reported the age of the patients included, though the groups were matched for age. One study with 16 AAA cases and 8 controls matched for sex [42]. Control abdominal aortic samples were collected from various sources, including transplant donors [42,43] or non-aneurysmal patients who underwent aortic surgery [77]. All three studies reported significantly higher IL-17 expression levels in AAA compared to control samples detected using Western blotting [43,77] or a multiplex cytokine panel assay [42]. A pooled analysis including 28 AAA cases and 20 controls suggested that IL-17 levels were significantly higher in AAA cases as compared to controls (Figure 2F). Quality assessment suggested a high risk of bias (Table 6). A rationale for the sample size was not provided, assessors were not blinded during cytokine measurements and cases or controls were not imaged.

Two studies measured IL-17 expression levels in serum samples from 629 AAA cases and 321 age- and sex-matched controls using ELISA [78,79]. One study reported significantly higher IL-17 levels in AAA cases by comparison with controls [79], whereas the other study reported significantly lower IL-17 serum levels in AAA cases compared to controls. IL-17 levels were positively correlated with aortic diameter size after adjusting for confounding factors in a multivariate analysis [78]. A pooled analysis including 629 AAA cases and 321 controls suggested that IL-17 was not significantly higher in AAA cases as compared to controls (Figure 2G). Quality assessment found one study adjusted for comorbidities and blinded the assessor during cytokine measurement [78]. Both studies reported that aortic imaging was performed using ultrasound [79] or computed tomography [78].

## 5. Safety Considerations for the Use of bDMARDs

AAA patients would most likely have to be on bDMARDs for an extended period of time if these drugs were to be used as a therapy to limit AAA growth and rupture. One of the major reasons for the withdrawal of bDMARDs is safety concerns due to their potent immuno-modulating abilities [90]. These drugs generally cause an increased risk of infections, which is why pneumococcal and influenza vaccines are suggested to be administered prior to bDMARD treatment initiation [91]. Due to the effect of this class of drugs on the immune system, live vaccines are to be used with extreme caution due to potential drug interactions in patients undergoing bDMARD therapy [92]. Specifically, patients undertaking bDMARDs are contraindicated for the administration of Herpes zoster vaccines due to the presence of live attenuated viruses [92]. In line with this suggestion, the Food and Drug Administration (FDA) also advises to evaluate active infections and tuberculosis (TB) in all patients for whom bDMARDs are considered (Table 7). Given that these are a relatively new class of drugs with limited safety information being available from clinical trials, the FDA advises that the manufacturers provide a ‘‘black box’’ warning in the labelling of these drugs. The specific safety warnings and contraindications of each bDMARD drug provided by the FDA are summarized in Table 7.

Safety data on bDMARDs in AAA patients are limited to one RCT using Canakinumab, an IL-1β inhibitor (ClinicalTrials.gov Identifier: NCT02007252). The wse of Canakinumab (n = 31) and placebo (n = 33) led to two (6.45%) compared with zero (0%) serious adverse events (SAE) over a period of one year. One case of stage 0 bladder cancer was reported in the intervention group. Sixteen infections and infestations were reported in the intervention group as compared to eleven cases in the placebo group (unpublished work [93]). No cases of cancer, serious infections or death were reported in either group. These results should be considered with caution for a number of reasons, including that the trial was terminated early, and there was a short treatment period and a small study population.

Previously, the European Union League Against Rheumatoid Arthritis (EULAR) recommended that all currently approved bDMARDs should be considered to have similar safety profiles [94]. A recent systematic literature review suggested that bDMARDs have a good safety profile [95]. To date, there are very limited 10-year post-marketing surveillance data for most bDMARDs owing to their recent arrival. One study analysed the adverse event rate following 12 years of Adalimumab (TNF inhibitor) exposure from 71 global clinical trials in 23,458 patients with rheumatoid arthritis (RA), juvenile idiopathic arthritis (JIA), ankylosing spondylitis (AS), psoriatic arthritis, psoriasis (Ps) or Crohn’s disease (CD) [96]. The study suggested that the safety profile of Adalimumab was consistent with the known information, and no new safety signals were raised [96]. A considerable number of studies have analysed several bDMARDs and reported good tolerability and an acceptable safety profile following five-year use. This includes analysis of Adalimumab, Infliximab and Etanercept (TNF inhibitors) in psoriatic arthritis [97], Ixekizumab (IL-17A monoclonal antibody) in moderate-to-severe psoriasis [98], Secukinumab (IL-17A monoclonal antibody) in moderate-to-severe plaque psoriasis, psoriatic arthritis and ankylosing spondylitis [99,100], Canakinumab (IL-1β monoclonal antibody) in systemic JIA [101], Sarilumab (IL-6 receptor) in rheumatoid arthritis [102] and Tildrakizumab (IL23 monoclonal antibody) in moderate-to-severe psoriasis [103].

Cancer is an anticipated risk with the use of bDMARDs. However, a recent two-year population-based study from a Taiwanese population suggested a significant association between reduced risk of all cancer and solid cancer with the use of Etanercept in 1111 RA patients by comparison to 16,812 RA patients who were naïve to bDMARDs [104]. These results were further supported by an analysis of the Australian Rheumatology Association Database’s (ARAD) prospective cohort study in which RA patients were treated with TNF inhibitors. This study suggested a lower incidence of malignancies compared to RA patients who were naïve to bDMARDs [105]. Similar results were reported from the United Kingdom from the British Society for Rheumatology Biologics Register for Rheumatoid Arthritis. There was no increased risk of cancer seen with any of the individual TNF inhibitors in RA patients when bDMARDs were added to the previously existing conventional DMARD therapy [106]. Solomon et al. reported that the cancer risk was elevated in patients receiving conventional DMARDs as compared to TNF inhibitors [107]. In fact, a long-term prospective observational study from 3529 Etanercept-treated patients reported improved survival, reduced cardiovascular events and reduced lymphoproliferative malignancies in comparison to 2864 conventional DMARD-treated patients [108]. A recent Danish population-based cohort study in 15,286 RA patients registered with the Danish Rheumatology database also suggested that treatment with bDMARDs was not associated with an increased risk of a second malignant neoplasm among patients with a history of cancer. It should be noted that the safety of bDMARDs in AAA patients who have high rates of concurrent malignancies is totally unknown.

## 6. Conclusions

Several preclinical and clinical studies have reported evidence implicating TNF and IL in AAA pathogenesis. bDMARDs that target these cytokines could potentially limit AAA progression, although this has not been adequately tested. The safety of long-term bDMARDs in people with AAA is not currently clear.

## Figures and Tables

**Table 1:** List of biologic DMARD drugs and their indications for use.

Drug Name	Trade Name	Specificity	Route of Administration	Indications for Use
**Tumor Necrosis Factor inhibitors**				
Etanercept [13]	ENBREL	sTNF, tmTNF, lymphotoxin A	SC injection	RA, JIA, PsA, AS, PPs, paediatric PPs
Infliximab [14]	REMICADE	sTNF, tmTNF	IV injection	RA, PJIA, AS, PsA, psoriasis, CD, paediatric CD, UC
Adalimumab [15]	HUMIRA	sTNF, tmTNF	SC injection	RA, adult and paediatric CD, UC, paediatric UC, AS, PsA, psoriasis
Certolizumab pegol [16]	CIMZIA	sTNF, tmTNF	SC injection	RA
Golimumab [17]	SYMPONI	sTNF, tmTNF	SC injection	RA, AS, PsA
**Interleukin inhibitors**				
Canakinumab [18]	ILARIS, previously ACZ885	IL-1β	SC injection	CAPS, FCAS, MWS, TRAPS, HIDS/MKD, Familial Mediterranean Fever
Anakinra [19]	KINERET	IL-1 Receptor A	SC injection	Moderate-to-severe active RA in patients 18 years of age or older who have failed one or more DMARDs
Tocilizumab [20]	ACTEMRA	IL-6	IV or SC injection	RA, pJIA and sJIA, Tocilizumab may be used alone or in combination with methotrexate; and in RA, other DMARDs may be used
Sarilumab [21]	KEVZARA	IL-6 Receptor	SC injection	For patients with moderate-to-severe active RA who have had an inadequate response or intolerance to one or more DMARDs
Ustekinumab [22]	STELARA	IL-12/23	IV or SC injection	Moderate-to-severe plaque psoriasis, active PsA, moderately to severely active CD
Guselkumab [23]	TREMFYA	IL-23	SC injection	Moderate-to-severe plaque psoriasis candidates for systemic therapy or phototherapy
Tildrakizumab [24]	ILUMYA	IL-23	SC injection	Moderate-to-severe plaque psoriasis patients who are candidates for systemic therapy or phototherapy
Secukinumab [25]	COSENTYX	IL-17A	SC injection	Moderate-to-severe plaque psoriasis patients who are candidates for systemic therapy or phototherapy, PsA, AS
Brodalumab [26]	SILIQ	IL-17 Receptor A	SC injection	Moderate-to-severe plaque psoriasis in adult patients who are candidates for systemic therapy or phototherapy and who have failed to respond or have lost response to other systemic therapies
Ixekizumab [27]	TALTZ	IL-17A	SC injection	Moderate-to-severe plaque psoriasis patients who are candidates for systemic therapy or phototherapy, active PsA

AS—ankylosing spondylitis, CD—Crohn’s disease, CAPS—cryopyrin-associated periodic syndromes, FCAS—familial cold autoinflammatory syndrome, HIDS—hyperimmunoglobulin D syndrome, IL—interleukin, IV—intravenous, JIA—juvenile idiopathic arthritis, MKD—mevalonate kinase deficiency, MWS—Muckle–Wells syndrome, PJIA—polyarticular juvenile idiopathic arthritis, PsA—psoriatic arthritis, PPs—plaque psoriasis, RA—rheumatoid arthritis, SC—subcutaneous, SJIA—systemic juvenile idiopathic arthritis, SC—subcutaneous, TNF—tumor necrosis factor, sTNF—soluble TNF, tmTNF—transmembrane TNF, TRAPS—TNF receptor-associated periodic syndrome, UC—ulcerative colitis.

**Table 2:** Examples of studies investigating the effect of inhibiting tumor necrosis factor or interleukins in AAA animal models.

Ref	Animal	AAA Model	Aortic Diameter (Intervention vs. AAA Control)	Intervention	Intervention Started after AAA Induction	Dose/Frequency of Intervention	Assessment Period	Post-Intervention Cytokine Change	*p* Value (TNF or IL Inhibition vs. AAA Controls)	Mechanisms Implicated in Protection from AAA Development or Growth
**Tumor Necrosis Factor-α**										
[28]	B6129SF2 mice	Periaortic application of CaCl_2_	9.8 ± 0.3 vs. 5.8 ± 0.1 mm	TNF alpha gene knockout Infliximab	NA Yes	NA 10 μg/g body weight, once weekly	6 weeks 6 weeks	↓ ↓	<0.01 0.03	Reduced elastic fiber disruption, macrophage infiltration, and MMP-2 and MMP-9 expression in aortic tissue
[29]	Mx-1 Cre transgenic mice	Periaortic application of CaCl_2_	1.3 ± 0.1 vs. 0.8 ± 0.1 mm ^	TACE gene knockout	No	250 μg on alternate days, starting 2 weeks prior to the operation	6 weeks	↓	0.05	Attenuated inflammation, oxidative stress, neoangiogenesis and extracellular matrix disruption
[30]	WKY	Elastase perfusion	2.7 ± 0.1 vs. 1.4 ± 0.1 mm	TNF-BP	No	1 mg/kg diluted in vehicle prior, 48 & 96 h	6 days	↓	<0.01	Elastin fragmentation and smooth muscle cell loss in the media of the aortic wall was prevented
**Interleukin-1b/1R**										
[30]	WKY	Elastase perfusion	2.3 ± 0.2 vs. 2.2 ± 0.3 mm	IL-1R-a	No	Dose: 100 mg/kg diluted in vehicle Frequency: 20 min prior to surgery, and every 8 h	6 days	↑	>0.05	NA
[32]	C57BL/6J mice	Periaortic application of CaCl_2_	58.2 ± 5.2 vs. 35.5 ± 3.5% ^	Genetic deletion of IL1β	No	NA	6 weeks	↓	0.01	NA
[34]	C57BL/6J mice	Ang-II infusion + IL-1Ra-deficient mice	0.9 ± 0.1 vs. 0.5 ± 0.0 mm	IL-1β mAb	Yes	7.5 mg/kg, twice a week	14 days	↓	<0.01	Prevented destruction of the elastic lamina and degeneration of SMCs in the abdominal aorta
[44]	C57BL/6J mice	Elastase perfusion	110% increase in AAA cases vs. self-controls	IL-1β knockout	No	NA	3, 7 and 14 days	↓	0.05	Attenuated ceramide synthesis in aortic infiltrated neutrophils prevents NETosis
[33]	C57BL/6J mice	Ang-II infusion + SMC selective Smad4 deletion in IL1-R1^−/−^	1.2 ± 0.0 vs. 1.5 ± 0.1 mm	IL-1β antibody	No	10 mg/kg body/weight, once weekly	16 weeks	↓	<0.01	Monocyte infiltration was blocked and aneurysm progression ameliorated
[35]	C57BL/6J mice	Elastase perfusion + IL-1β gene knockout	38 ± 20.4 vs. 89.5 ± 13.1% 52.9 ± 3.2 vs. 82.4 ± 15.3%	IL-1R gene knockout IL-1R antagonist (anakinra)	No Yes	Anakinra administered at day 3 post-AAA induction at 100 mg/kg per day	14 days	NA	NA	Decreased macrophage and elastin fragmentation
**Interleukin-6**										
[37]	C57BL/6J mice	Periaortic application of CaCl_2_	0.9 ± 0.0 vs. 1.1 ± 0.0 mm	murine anti-IL-6R	Prior and post induction	0.25 mg MR16-1 every week	6 weeks	↓	<0.01	Suppressed STAT3 activation and AAA expansion
[36]	C57BL/6J mice	Elastase perfusion	50 ± 20.9 vs. 82.7 ± 25.1 mm ^	Anti-IL-6 antibody	Yes	4 mg/kg, initiated at day 3	14 days	↓	<0.03	Reduced AAA progression
[39]	C57BL/6J mice	Elastase perfusion	101.2 ± 20.1 vs. 101.2 ± 18.4% ^	IL-6 knockout	No	NA	14 days	↔ (Unchanged)	0.73	NA
[38]	C57BL/6J mice	elastase + anti-TGF-β model	1.6 ± 0.3 vs. 1.9 ± 0.5	sgp130Fc	Yes	10µg thrice a week initiated on the day of experiment	7 days	↓	<0.01	Increased collagen content of the arterial wall
**Interleukin-12/23**										
[40]	C57BL/6J mice	Ang-II infusion	1.4 ± 0.1 vs. 1.1 ± 0.1 mm ^	IL-12p40 knockout	No	150 μL 2 timesat 3-day interval	14 days	↓	<0.01	Augmented TGFβ2-mediated MMP2 expression
[41]	C57BL/6J mice	Elastase perfusion	0.5 ± 0.1 vs. 0.7 ± 0.1 mm	IL-12p40/IL-23p19 mAb	Yes	250 μg on days 3 and 8	14 days	↓	<0.001	Reduced M1 and M2 macrophages
**Interleukin-17**										
[43]	ApoE^−/−^ mice	Ang-II infusion	1.4 ± 0.1 vs. 1.7 ± 0.1 mm	IL-17A siRNA	No	3μg/kg	28 days	↓	0.05	Reduced VEGFA, MMP-2, MMP-9 and JAK2 protein levels.
[42]	C57BL/6J mice	Elastase perfusion	89.4 ± 7.4 vs. 141.1 ± 16.1%	IL-17^−/−^	No	NA	14 days	↓	<0.05	Reduced MCP-1, RANTES, KC, TNF-α, MIP-1α and IFN-γ

AAA—abdominal aortic aneurysm, ApoE—apolipoprotein E, Ang-II—angiotensin-II, CaCl_2_—calcium chloride, CD—cluster of differentiation, ECM—extracellular matrix, IFNγ—interferon gamma, IL—interleukin, JAK—Janus kinase, kg—kilogram, KC—keratinocyte-derived chemokine, MMP—matrix metalloproteinases, MCP—monocyte chemoattractant protein, MSC—mesenchymal stem cells, MIP1α—macrophage inflammatory protein 1 alpha, mAb—monoclonal antibody, μL—microlitre, mg—milligram, μg—microgram, NA—not available; NR—not reported, ND—non-detectable, NETosis—neutrophil extracellular traps, RANTES—regulated upon activation normal T cell expressed and presumably secreted, STAT—signal transducer and activator of transcription, SMAD4—mothers against decapentaplegic homolog 4, siRNA—small interfering ribonucleic acid, SMC—smooth muscle cell, TNFα—tumor necrosis factor alpha, TN-BP—TNF binding protein, TACE—TNF-alpha converting enzyme, TGFβ—transforming growth factor beta, VEGF—vascular endothelial growth factor, WKY—Wistar–Kyoto, IL-1R-a—interleukin 1 receptor a, %—percentage. ^ Mean ± SD of aortic diameter calculated using ImageJ [45] for graphs and using a validated method [46] for median values.

**Table 3:** Quality of animal studies investigating the effect of tumor necrosis factor or interleukin inhibition in AAA models.

Ref	Ethics Approval	Animal Strain and Number	Animal Age/Weight	AAA Model	Controls Used	Aortic Diameter	AAA Measurement Methods	Reproducibility of Measurements	Randomisation	Blinding of Assessors
[28]	Yes	Yes	Yes	Yes	Yes	No	No	No	No	No
[29]	Yes	Yes	Yes	Yes	Yes	Yes	No	No	No	No
[30]	Yes	Yes	Yes	Yes	Yes	Yes	Yes	Yes	No	No
[32]	Yes	Yes	No	Yes	Yes	No	Yes	No	No	No
[34]	Yes	Yes	No	Yes	Yes	Yes	Yes	No	No	No
[44]	Yes	Yes	No	Yes	Yes	No	Yes	No	No	No
[33]	Yes	Yes	Yes	Yes	Yes	Yes	Yes	No	No	No
[35]	Yes	Yes	Yes	Yes	Yes	Yes	Yes	Yes	No	Yes
[37]	Yes	Yes	Yes	Yes	Yes	Yes	Yes	No	No	No
[36]	Yes	Yes	Yes	Yes	Yes	No	Yes	No	No	Yes
[39]	Yes	Yes	Yes	Yes	Yes	No	Yes	No	No	No
[38]	Yes	Yes	Yes	Yes	Yes	No	Yes	No	No	No
[40]	Yes	Yes	Yes	Yes	Yes	No	Yes	No	No	No
[41]	Yes	Yes	Yes	Yes	Yes	Yes	Yes	No	No	Yes
[43]	Yes	Yes	Yes	Yes	Yes	Yes	Yes	No	No	No
[42]	Yes	Yes	Yes	Yes	Yes	Yes	Yes	No	No	No

AAA—abdominal aortic aneurysm.

**Table 4:** Examples of clinical studies comparing tumor necrosis factor or interleukin expression levels in AAA and control tissue samples.

Ref	Number of AAA vs. Control Cases	Age of AAA vs. Control Cases (*p* Value), Years	Male Gender % (AAA vs. Control Cases)	Aortic Diameter in AAA (mm)	Method of Assessment	Cytokine Concentration in AAA Cases	Cytokine Concentration in Control Cases	*p* Value
**Tumor Necrosis Factor-α**								
[56]	32 vs. 11	70.5 ± 7.5 vs. 59.5 ± 4.5	74.4 vs. 75 ⁿ	≥50 mm	IHC	15.0 ± 22.9 pg/mg	9.6 ± 9.2 pg/mg	<0.002
[50]	27 vs. 5	NR	NR	NR	IHC	5.5 ± 3.5 pg/mg	0.6 ± 0.63 pg/mg	<0.05
[51]	10 vs. 9	73 (67–81) vs. 55(44–74) ^x^	100 vs. 66.7	75 (56 to 93) mm ^x^	Antibody based protein array	60 ± 20 × 10^−3^ (SI)	2 ± 3 × 10^−3^ (SI)	<0.01
[61]	17 vs. 11	72.4 ± 6.2 vs. 55.6 ± 10.2	82.3 vs. 63.6	6.7 ± 1.1 cm	RT-PCR	0.2 (0.0–0.8)	ND	<0.01
[53]	15 vs. 6	70 ± 6 vs. 41 ± 14	NR	NR	ELISA	24 ± 11 pg/mL	13 ± 1.5 pg/mL	NS
[52]	7 vs. 5	NR	NR	NR	ELISA	86 ± 34 pg/mg	1 ± 1 pg/mg	<0.01
[49]	8 vs. 8	64.8 ± 2.9 vs. 60.8 ± 3.6	87.5 vs. 100	NR	ELISA	10 ± 1.6 ng/mL ^	6.68 ± 2.05 ng/mL ^	<0.05
[55]	82 vs. 73	73 (50–88) vs. 62 (43–82) (*p* < 0.01) ^x^	90 vs. 85	>5 cm	RT-PCR	5.6 ± 2.7 × 10^−4^ am/μL	4.4 ± 2.7 × 10^−5^ am/μL	<0.01
[83]	6 vs. 7	66.8 ± 5.9 vs. 62 ± 14.5 ᵇ	60 vs. 70	NR	Affymetrix and illumina microarray	Relative expression to controls—0.65		<0.05
[54]	19 vs. 5	72 ± 6 vs. 46 ± 4	89.4 vs. 80	NR	RT-PCR	2.8 ± 0.5 (GAPDH ratio)	1.0 ± 0.1 (GAPDH ratio)	<0.05
**Interleukin-1β/1R**								
[56]	32 vs. 11	70.5 ± 7.5 vs. 59.5 ± 4.5	74.4 vs. 75 ⁿ	≥50 mm	IHC	34.5 ± 37.5 pg/mg	4.8 ± 2.7 pg/mg	<0.01
[51]	10 vs. 9	73 (67–81) vs. 55(44–74) ^x^	100 vs. 66.7	75 (56 to 93) mm	Antibody based protein array	upregulated	ND	<0.01
[61]	17 vs. 11	72.4 ± 6.2 vs. 55.6 ± 10.2	82.3 vs. 63.6	6.7 ± 1.1 cm	RT-PCR	7.2 ± 5.01 pg/mg ^x^	0.71 ± 0.60 pg/mg ^x^	<0.01
[53]	15 vs. 6	70 ± 6 vs. 41 ± 14	NR	NR	ELISA	908 ± 194 pg/mL	100 ± 30 pg/mL	0.05
[52]	7 vs. 5	NR	NR	NR	ELISA	48 ± 14 pg/mg	12 ± 5 pg/mg	<0.05
[49]	8 vs. 8	64.8 ± 2.9 vs. 60.8 ± 3.6	87.5 vs. 100	NR	ELISA	14.5 ± 2.9 ng/mL ^	10.2 ± 1.8 ng/mL ^	<0.05
[83]	6 vs. 7	66.8 ± 5.9 vs. 62 ± 14.5 ᵇ	60 vs. 70	NR	Affymetrix and illumine microarray	Relative expression to controls—1.6		<0.01
[60]	20 vs. 4	77.3 vs. 60.5 ^	80 vs. 50	7.5 (5–10) cm	ELISA	91 ± 23.6 pg/mg	60.3 ± 12.1 pg/mg	NS
[84]	10 vs. 10	75 (61–82) ^x^	NR	>5 cm	Affymetrix Human Genome microarray	Fold change—3.94		0.05
**Interleukin-6**								
[56]	32 vs. 11	70.5 ± 7.5 vs. 59.5 ± 4.5	74.4 vs. 75 ⁿ	≥50 mm	IHC	527.9 ± 468.9 ng/mL	28.3 ± 13.9 ng/mL	<0.01
[51]	10 vs. 9	73 (67–81) vs. 55(44–74) ^x^	100 vs. 66.7	75 (56–93) mm	Antibody based protein array	150 ± 500 × 10^−3^ (SI)	10 ± 10 × 10^−3^ (SI)	<0.01
[49]	8 vs. 8	64.8 ± 2.9 vs. 60.8 ± 3.6	87.5 vs. 100	NR	ELISA	5.5 ± 2.15 ng/mL ^	1.7 ± 0.4 ng/mL ^	<0.05
[55]	82 vs. 73	73 (50–88) vs. 62 (43–82) (<0.01) ^x^	90 vs. 85	>5 cm	RT-PCR	0.6 ± 0.4 am/μL	0.01 ± 0.01 am/μL	0.02
[60]	20 vs. 4	77.3 vs. 60.5 ^	80 vs. 50	7.5 (5–10) cm	ELISA	90.3 ± 31.7 ng/mL	28.1 ± 5.9 ng/mL	NS
[63]	7 vs. 4	NR	NR	NR	ELISA	1008 ± 197 ng/mL	190 ± 14 ng/mL	<0.05
[62]	13 vs. 16	NR	NR	NR	ELISA	14,329 ± 4159 U/mL	1861 ± 334 U/mL	0.02
[84]	10 vs. 10	75 (61–82) ^x^	NR	>5 cm	Affymetrix Human Genome microarray	Fold change—6.9		<0.05
**Interleukin-12/23**								
[51]	10 vs. 9	73 (67–81) vs. 55(44–74) ^x^	100 vs. 66.7	75 (56 to 93) mm	Antibody based protein array	0.04 [0.01 to 0.07] ^x^	0.00 [0.00 to 0.04] ^x^	0.02
[61]	17 vs. 11	72.4 ± 6.2 vs. 55.6 ± 10.2	82.3 vs. 63.6	6.7 ± 1.1 cm	RT-PCR	0.05 (0–0.3) ^x^	ND	NS
[83]	6 vs. 7	66.8 ± 5.9 vs. 62 ± 14.5 ᵇ	60 vs. 70	NR	Affymetrix and illumina microarray	Relative expression to controls—1.3		<0.01
**Interleukin-17**								
[43]	7 vs. 7	NR	NR	NR	Western blotting	0.5 ± 0.08	0.03 ± 0.03	<0.01
[42]	16 vs. 8	NR	100 vs. 100	NR	Multiplex cytokine panel assay	37.8 ± 3.8 pg/mL	6.0 ± 3.2 pg/mL	< 0.05
[77]	5 vs. 5	NR	NR	NR	Western blotting	0.7 ± 0.2	0.07 ± 0.04	<0.01

All data presented as mean ± SD. Mean was calculated from the graphical data using ImageJ [45]; if median data were provided, mean was calculated using a validated method [46]. ᵇ Age calculated from raw data provided within the study. ⁿ Gender was allocated in 3:1 male-to-female ratio and therefore assumed as 75% male. ^ Standard deviation not reported or calculated from the graph. ^x^ Median value provided in the study. AAA—abdominal aortic aneurysm, am—atomic moles, cm—centimetre, ELISA—enzyme-linked immunosorbent assay, GAPDH—glyceraldehyde-3-phosphate dehydrogenase, IHC—immunohistochemistry, mg—milligram, mm—millimetre, μL—microlitre, ng—nanogram, NR—not reported, NS—not significant, ND—not detectable, pg—picogram, RT-PCR- reverse transcriptase polymerase chain reaction, SI—signal intensity, %—percentage.

**Table 5:** Quality assessment of the clinical studies investigating tumor necrosis factor or interleukins.

Ref	Sample Size Estimate Reported	Age-Matched Controls	Sex-Matched Controls	Comorbidities Were Adjusted for in Analyses	Analysis by Blinded Observer	Controls and AAA Cases Imaged	Method and Mode of Aortic Diameter Imaging
[43]	No	No	No	No	No	No	NA
[42]	No	No	No	No	No	No	NA
[56]	No	Yes	Yes	No	No	No	NA
[50]	No	NR	NR	No	No	No	NA
[51]	No	Yes	Yes	No	No	No	NA
[61]	No	Yes	Yes	No	No	No	NA
[53]	No	Yes	NR	No	No	No	NA
[52]	No	NR	NR	No	No	No	NA
[49]	No	Yes	Yes	No	No	No	NA
[55]	No	Yes	Yes	No	No	No	NA
[83]	No	Yes	Yes	No	No	No	NA
[54]	No	Yes	Yes	No	No	No	NA
[59]	No	Yes	Yes	No	No	Yes	Ultrasonography *
[57]	Yes	Yes	Yes	No	No	Yes	Ultrasound *
[60]	No	Yes	Yes	No	No	No	NA
[84]	No	Yes	Yes	No	No	No	NA
[63]	No	NR	NR	No	No	No	NA
[62]	No	NR	NR	No	No	No	NA
[75]	No	Yes	Yes	No	No	Yes	Ultrasonography *
[65]	No	Yes	Yes	No	Yes	Yes	Computed tomography **
[66]	No	Yes	Yes	No	No	Yes	Ultrasound *
[73]	No	Yes	Yes	No	No	Yes	Ultrasonography *
[64]	No	Yes	Yes	No	No	Yes	Ultrasound *
[67]	No	Yes	Yes	No	No	Yes	Ultrasound or computed tomography ^#^
[72]	No	No	No	No	No	No	NA
[58]	Yes	Yes	Yes	No	No	Yes	Ultrasonography *
[85]	No	Yes	Yes	No	No	Yes	Ultrasonography *
[69]	No	Yes	No	No	No	No	NA
[70]	No	Yes	Yes	No	No	Yes	Ultrasonography *
[71]	Yes	Yes	Yes	Yes	No	Yes	Ultrasonography *
[76]	No	Yes	Yes	Yes	No	Yes	Ultrasonography *
[74]	No	No	No	No	No	No	NA
[68]	No	Yes	Yes	No	No	Yes	Contrast-enhanced computed tomography
[77]	No	No	No	No	No	No	NA
[79]	No	Yes	Yes	No	No	Yes	Ultrasound *
[78]	No	Yes	Yes	Yes	Yes	Yes	Computed tomography *

* Aortic diameter imaged. ** Thrombus content imaged/estimated; NA—not applicable. ^#^ Imaging performed within two years from start of the study.

**Table 6:** Examples of clinical studies comparing circulating tumor necrosis factor or interleukin serum concentrations in AAA and control participants.

Ref	Number of AAA vs. Control Cases	Age of AAA vs. Control Cases (*p* Value), Years	Male Gender % (AAA vs. Control Cases)	Aortic Diameter in AAA (mm)	Method of Assessment	Cytokine Concentration in AAA Cases	Cytokine Concentration in Control Cases	*p* Value
**Tumor Necrosis Factor-α**								
[59]	50 vs. 42	58.6 ± 6.6 vs. 58.1 ± 6.3 *	80 vs. 44.7	48 (33–66) mm	Solid phase radioimmunoassay	2.1 ± 0.7 pmol/L ^	1.5 ± 0.3 pmol/L ^	<0.05
[57]	20 vs. 20	74 ± 6 vs. 72 ± 5	100 vs. 100	<45 mm	ELISA	41.4 ± 17.3 pg/mL	13.1 ± 5.2 pg/mL	<0.05
[58]	130 vs. 219 ^z^	75 ± 8 vs. 68 (53–80)	82.6 vs. 90	>55 mm	ELISA	3.6 ± 10.2 pg/mL	1.23 ± 1.93 pg/mL	<0.01
**Interleukin-1β**								
[59]	50 vs. 42	58.6 ± 6.6 vs. 58.05 ± 6.3 *	80 vs. 44.7	48 (33–66) mm	Solid phase radioimmunoassay	19.3 pmol/L	2.1 pmol/L	<0.01
**Interleukin-6**								
[59]	50 vs. 42	58.6 ± 6.6 vs. 58.05 ± 6.3 *	80 vs. 44.7	48 (33–66) mm	Solid phase radioimmunoassay	10.0 ± 5.3 pmol/L ^	4.8 ± 3.2 pmol/L ^	<0.05
[75]	38 vs. 41	70(66–76) vs. 72(67–79) ^x^	71 vs. 80.5	4.0 (3.5–4.3) cm	ELISA	3.6 ± 0.51 pg/mL ^x^	3.0 ± 1.03 pg/mL ^x^	NS
[65]	27 vs. 15	73 (58–91) vs. 50 (32–74) (*p* < 0.01) ^x^	100 vs. 20	64 (51–100) mm	ELISA	4.9 ± 0.4 pg/mL	2.6 ± 0.5 pg/mL	<0.05
[66]	89 vs. 98	73.5 ± 0.5 vs. 73.5 ± 0.5	71.9 vs. 71.4	4.5 (3.9 to 5.1) cm	ELISA	2.9 ± 0.4 pg/mL ^x^	1.9 ± 0.2 pg/mL ^x^	<0.05
[73]	74 vs. 30	70.7 (56–82) vs. NR	80 vs. NR	5 (5–8), vs. NR cm	ELISA	64.2 ± 15.7 pg/mL	6.7 ± 5.1 pg/mL	<0.05
[64]	214 vs. 343	74 ± 8 vs. 68 ± 2 (*p* < 0.01)	79 vs. 46.3 (*p* < 0.01)	62.8 ± 14.6 mm	ELISA	9.4 ± 32.2 pg/mL	2.1 ± 2.9 pg/mL	<0.01
[67]	108 vs. 42	72 ± 4 vs. 69 ± 8	100 vs. 100	6.3 ± 0.8 cm	Immunoassay	5.5 ± 2.4 pg/mL	4.2 ± 1.6 pg/mL	0.04
[72]	41 vs. 18	72.0 (63.4–77.8) vs. 59.6 (51.4–69.4)	92.7 vs. 55.6	61.6 (40–112) mm	ELISA	3.7 ± 0.6 pg/mL ^x^	3.1 ± 0.9 pg/mL ^x^	NS
[58]	130 vs. 219 ^z^	75 ± 8 vs. 68 (53–80) ^x^	82.6 vs. 90	>55 mm	ELISA	11.3 ± 33.8 pg/mL	2.1 ± 2.9 pg/mL	<0.01
[85]	23 vs. 20	72 (54–83) vs. 72 (66–79)	100 vs. 80	60 (43–75) ^x^ mm	ELISA	940 ^x^ ng/mL	793 ^x^ ng/mL	<0.01
[69]	10 vs. 10	72 (62–75) vs. 72 (62–75)	80 vs. 20	NR	ELISA	12.4 ± 11.2 pg/mL	3.4 ± 3.1 pg/mL	0.02
[70]	78 vs. 36	71 (66–78) vs. 72 (67–78)	79.5 vs. 83.3	49 (40–61) ^x^ mm	ELISA	3.1 ± 0.6 ng/mL	2.3 ± 0.4 ng/mL	<0.01
[71]	75 vs. 90	72 ± 7 vs. 72 ± 6	100 vs. 100	41 (35–46) mm	ELISA	3.1 ±0.2 ^x^ ng/mL	3.1 ± 0.2 ^x^ ng/mL	0.98
[76]	7 vs. 113	65 ± 9 (both groups combined)	52.5 vs. 67.5	2.1 ± 0.6 cm/m^2^	ELISA	3.2 ± 0.9 pg/mL	2.3 ± 1.2 pg/mL	0.04
[74]	50 vs. 22	72.0 (54–85) vs. 59.6 (44–78)	90 vs. 54.5	61.6 (40–112) mm	ELISA	4 ± 0.6 ^x^ pg/mL	2.7 ± 0.5 ^x^ pg/mL	<0.01
[68]	10 vs. 10	76.5 (65–85) vs. 70.5 (59–81)	80 vs. 80	56.1 (48–83) mm	ELISA	22.6 ± 13.7 ^x^ pg/mL	9.8 ± 5.6 ^x^ pg/mL	<0.05
**Interleukin-17**								
[79]	153 vs. 121	68.9 ± 4.9 vs. 69.4 ± 6.4	96.7 vs. 99.2	49.4 mm ^	ELISA	27.7 ± 42.1 pg/mL	11.7 ± 25.1 pg/mL	<0.01
[78]	476 vs. 200	69.9± 2.8 vs. 69.6 ± 2.8	100 vs. 100	50 mm	ELISA	36.5 ± 5.9 pg/mL	68.4 ± 13.3 pg/mL	0.02

All data presented as mean ± SD. Mean was calculated from the graphical data using ImageJ [45]; if median data were provided, data was calculated using a validated method [46]. * Mean age was calculated using data provided for both male and female genders separately. ^ Standard deviation not reported. ^x^ Median value provided in the original paper. ^z^ AAA data were reported separately as small <45 mm (n = 122), medium 45–55 mm (n = 108) and large >55 mm (n = 130). Large aneurysm (>55 mm) group was selected for pooled analysis. AAA—abdominal aortic aneurysm, cm—centimetre, ELISA—enzyme-linked immunosorbent assay, L—litre, mL—millilitre, mm—millimetre, NR—not reported, NS—not significant, ND—not detectable, pmol—picomole, pg—picogram.

**Table 7:** Safety considerations for the use of bDMARDs reported by the Food and Drug Administration.

Drug Class	Safety Warnings from FDA	Contraindications
Infliximab [14]	Increases mortality in moderate or severe heart failure (NYHA class III/IV) Increased cases of tuberculosis and other serious infections including histoplasmosis, listeriosis and pneumocytosis Increased risk of acute liver failure, jaundice, hepatitis and cholestasis Increased risk of malignancies Hypersensitivity to the active substance	Moderate-to-severe heart failure Hypersensitivity to Infliximab or inactive components or to any murine proteins
Etanercept [13]	Nervous system disorders including demyelinating disorders such as multiple sclerosis, myelitis and optic neuritis Active infections Hypersensitivity to the active substance	Sepsis
Adalimumab [15]	Serious infections when administered in combination with Anakinra Hypersensitivity reactions Hematologic events including pancytopenia and aplastic anaemia	None
Certolizumab pegol [16]	Do not start during serious infection, e.g., invasive fungal infection Monitor for worsening or new onset heart failure or demyelinating disease Monitor for cytopenia and lupus-like syndrome Hypersensitivity to the active substance	None
Golimumab [17]	Do not start during serious infection and malignancies Monitor for worsening or new onset heart failure or demyelinating disease Hepatitis B reactivation Hypersensitivity to the active substance	None
Canakinumab [18]	Activation of infection Hypersensitivity to the active substance	Combination with TNF inhibitors Hypersensitivity to Canakinumab or any of the inactive ingredients
Anakinra [19]	Serious infections Higher rate of infections and neutropenia is seen when used in combination with etanercept	Use of Anakinra with TNF-blocking agents should be given with extreme caution
Tocilizumab [20]	Serious infections leading to hospitalization or death, including tuberculosis, bacterial, invasive fungal, viral and other opportunistic infections Gastrointestinal perforation Monitor potential consequences of treatment-related changes in neutrophils, platelets, lipids and liver function tests Hypersensitivity	Hypersensitivity to Tocilizumab
Sarilumab [21]	Serious infections Monitor potential consequences of treatment-related changes in neutrophils, platelets, lipids and liver function tests Gastrointestinal perforation	Hypersensitivity to sarilumab or any of the inactive ingredients Active infections
Ustekizumab [22]	Serious infections Tuberculosis Malignancies Reversible posterior leukoencephalopathy syndrome	Hypersensitivity to Ustekizumab or any of the inactive ingredients Active infections
Guselkumab [23]	Evaluate infections and tuberculosis	None
Tildrakizumab [24]	Evaluate infections and tuberculosis	Serious hypersensitivity reaction to tildrakizumab or to any of the excipients
Secukinumab [25]	Evaluate oral candidiasis and malignancy	Serious hypersensitivity reaction to secukinumab or to any of the excipients
Brodalumab [26]	Depression, suicide ideation and behaviour disorders Serious infections, e.g., tuberculosis	Crohn’s disease
Ixekizumab [27]	Evaluate infections and tuberculosis	Serious hypersensitivity reaction to ixekizumab or to any of the excipients Inflammatory bowel disease: Crohn’s disease and ulcerative colitis including exacerbations

NYHA: New York Heart Association scale.

## Data Availability

All relevant data used for data analyses and interpretation are available within the main manuscript.
